# Cultural sensitivity and associated factors among nurses in southwest Ethiopia: a cross-sectional study

**DOI:** 10.1186/s12912-024-01838-8

**Published:** 2024-03-15

**Authors:** Robera Demissie Berhanu, Eba Abdisa Golja, Tesfaye Abera Gudeta, Jira Wakoya Feyisa, Dame Habtamu Rikitu, Yadeta Babu Bayane

**Affiliations:** 1 School of Nursing and Midwifery, Institute of Health Sciences, Wallaga University, Nekemte, P. O. Box: 395, Ethiopia; 2 Department of Public Health, Institute of Health Sciences, Wallaga University, Nekemte, Ethiopia; 3 Department of Obstetrics and Gynecology, College of Health Sciences, Addis Ababa University, Addis Ababa, Ethiopia; 4 Department of Clinical Pharmacy, Institute of Health Sciences, Wallaga University, Nekemte, Ethiopia

**Keywords:** Culture, Cultural sensitivity, Nurses, Ethiopia

## Abstract

**Background:**

Because of the rapidly rising cultural diversity, the ability to recognize cultural diversity is extremely important to all healthcare professionals, especially to nurses. However, there is a paucity of information regarding the cultural sensitivity of nurses in Ethiopia. Hence, this study aimed to assess cultural sensitivity and associated factors among nurses working at Jimma Medical Center, Oromia Regional State, Southwest Ethiopia.

**Methods:**

Health-facility-based cross-sectional study was conducted among 244 nurses selected by simple random sampling from May 20th to June 20th, 2020. A structured, self-administered questionnaire was used to collect data. Data were analysed using Statistical Product and Service Solution Version 26.0. Bivariate binary logistic regression analyses were used to select variables for the final model. Multivariable binary logistic regression analysis was used to identify factors associated with cultural sensitivity. Statistical significance was declared at $p\leq$0.05, and adjusted odds ratio with respective 95% CI was used to report significant covariates.

**Results:**

Out of the total sample, 236 nurses participated in this study, giving a response rate of 96.72%. Nurses who were culturally sensitive while delivering routine nursing services were found to be 40.3% (95% CI (34.3, 46.6)). Level of education ([AOR (95% CI)], [4.846 (1.188, 19.773)]), interpersonal communication ([AOR (95% CI)], [4.749 (1.334, 16.909)]), and intercultural communication ([AOR (95% CI)], [51.874 (13.768, 195.45)]) were positively and significantly associated with the cultural sensitivity of nurses.

**Conclusion:**

Cultural sensitivity is found to be low in the study area. Increasing level of education, effective interpersonal communication abilities, and intercultural communication abilities positively predict cultural sensitivity of nurses. It is helpful for nurses to improve their knowledge of transcultural nursing theories and cultural understanding.

**Supplementary Information:**

The online version contains supplementary material available at 10.1186/s12912-024-01838-8.

## Background

Nurses are expected to provide nursing care to patients with diverse cultural backgrounds by displaying a holistic and intercultural approach. In the 1960s, Madeleine M. Leininger, a nursing theorist, established transcultural nursing care as an area of study and practice by amalgamating the disciplines of nursing and anthropology [1]. The goal of transcultural nursing is to develop a body of knowledge that helps develop culture-universal and culture-specific nursing care [2], and her transcultural nursing theory recognizes the strong influence of culture on health and illness [3, 4]. Culture is defined as the learned and shared knowledge and symbols that specific groups of people use to interpret their experience of reality and to direct their thinking and behavior. A different way of beholding the world, people, relationships, and events that make up a culture may be unique to an ethnic group, or it may be a worldview that is shared by a nation [5].

Globally, cultural diversity is the leading cause of health disparities. Cultural diversity is resulting from globalization, immigration, and medical tourism across the globe [6–10]. Because of the rapidly rising cultural diversity, the ability to recognize cultural diversity is extremely important to all healthcare professionals, especially nurses, who come into very close contact with healthcare seekers of different cultures. Since nurses are increasingly forced to work with culturally diverse clients, they are required to understand the cultural care beliefs, values, and lifeways of patients. Nurses also need to immerse themselves in the culture of all patients to provide care that is consistent with what the patients perceive as appropriate based on their cultural expectations [1, 11, 12]. Therefore, nurses must be prepared to detect clients’ culturally derived desires and develop skills that will aid their achievement [13].

There are global concerns about health disparities since the cultural diversity of the population is increasing. Numerous studies indicate that health disparities continue to demonstrate evidence for healthcare providers to pay attention to cultural diversity [14, 15]. Consequently, the quality of care is affected by growing cultural diversity [16], and the common problems nurses face when dealing with patients from different cultural backgrounds are cultural barriers [17]. Since nurses lie at the front line of the healthcare system, they have the opportunity to stand out as the ones who can readily understand and identify the health issues of patients with different cultures [3, 11]. Evidence also indicates that cultural sensitivity is crucial for the provision of culturally competent care [18]. Cultural sensitivity is defined as cultivating good feelings for comprehending and examining cultural differences, and it highlights the various qualities people must possess to enhance cultural competency. These qualities include empathy, self-control, broad-mindedness, interactive relationships, and abstaining from bias and judgment [19, 20]. To meet the needs of all individuals with different cultures and to provide culturally competent care, nurses must have sufficient cultural sensitivity and incorporate it into nursing care [21]. Thus, it is very important to investigate the cultural sensitivity of nurses to ensure the quality of care for patients.

Today, each country is becoming more multicultural. However, a review of the literature has shown that there is a paucity of studies in Africa, particularly in Ethiopia, that have investigated the cultural sensitivity of nurses, and the consideration of cultural disparity is given less concern. Therefore, this study aimed to evaluate cultural sensitivity and associated factors among nurses working at Jimma Medical Center, Oromia Regional State, Southwest Ethiopia. The findings from this study help provide evidence-based nursing care to clients from varied cultural and ethnic backgrounds.

## Methods

### Study setting and population

Health facility-based cross-sectional study was conducted at Jimma Medical Center (JMC) between May 20 and June 20, 2020. JMC is based in Jimma City, which is located at 352 km from Ethiopia’s capital, Addis Ababa. About 15 to 20 million people receive various services from the hospital, including outpatient and inpatient treatment, maternal and child health services, referral and follow-up services, physiotherapy services, rehabilitative services, intensive care, and recovery services. At the time, the hospital had 553 nurses. The vast majority of these nurses (518) were actively working at JMC, while others (35) were on study leave. All JMC nurses who met the eligibility criteria were determined to be the study population. The study included nurses who had worked at JMC for at least six months. Nurses who were absent from work during the study period due to various reasons, including annual leave, maternity leave, or other social issues, were excluded.

### Sampling

Sample size was determined using a formula for a single population proportion, considering the following assumptions: 95% confidence interval (Z = 1.96), 5% margin of error (d = 0.05), and 50% population proportion (*p* = 0.5) since there is no similar study conducted on cultural sensitivity among nurses or factors affecting cultural sensitivity in Ethiopia. Based on the above-stated assumptions, sample size was calculated as follows:

$$ {n}_{i}=\frac{{\left({Z}_{\raisebox{1ex}{$\alpha $}\!\left/ \!\raisebox{-1ex}{$2$}\right.}\right)}^{2}\times p(1-p)}{{d}^{2}}$$

$$ {n}_{i}=\frac{{\left(1.96\right)}^{2}\times 0.5(1-0.5)}{{0.05}^{2}}$$

Correction formula was applied to get the final sample size because the total number of JMC nurses was fewer than 10,000 and the ratio of the initial sample size ($$ {n}_{i}$$) to the total number of nurses in JMC (N) was greater than 0.05. Accordingly, the minimum sample size required was calculated as follows:

$$ {n}_{f}=\frac{{n}_{i}}{1+ \frac{{n}_{i}}{N}}$$

$$ {n}_{f}=\frac{384}{1+ \frac{384}{518}} =\frac{384}{1+ 0.74} = \frac{384}{1.74} =221$$

Where;

- $$ {n}_{i}$$ is the initial sample size
- $$ {n}_{f}$$ is the final sample size
- $\text{N}$ is the number of nurses working at JMC

Finally, 10% non-response was added, and the final sample size became **244.**

Participants in the study were chosen using simple random sampling technique. First, a sampling frame was created depending on the list of all nurses obtained from JMC. Then, simple random sampling using lottery methods was employed to select the study participants.

### Data collection and instruments

Data were collected by four B.Sc. nurses working at another hospital other than JMC. The principal investigator made constant monitoring throughout the data collection process. Before actual data collection, the study instruments were pretested at Agaro General Hospital among 25 nurses. From the results of the pre-test, the internal consistency of the study instruments was measured, data collection time was estimated, and some modifications were made to the study instruments as needed. The internal consistency was tested using a reliability scale, and the results are presented in the table that follows [Table 1].

**Table 1:** Reliabilities of Each Scale used in this Research on Pre-test conducted among Nurses working at Agaro General Hospital, Oromia Region, Southwest Ethiopia, May 2020 (*n* = 25)

Variables	Number of items	Cronbach’s alpha
Intercultural communication scale	5	0.914
Cultural sensitivity scale	3	0.886
Interpersonal communication scale	7	0.936
Cultural motivation scale	5	0.943

Data were collected using structured, self-administered data collection tools that were adapted from related published studies [22–24]. The data collection tools had five parts: (a) socio-demographic questionnaire; (b) cultural sensitivity scale (CSS); (c) interpersonal communication scale (IPCS); (d) intercultural communication scale (ICS); and (e) cultural motivation scale (CMS).

#### Cultural Sensitivity Scale (CSS) and Intercultural Communication Scale (ICS)

CSS and ICS were previously developed and published online by Ulrey & Amason (2001) [24]. CSS is a 5-point Likert scale, which consists of 3 items asking about patients’ cultures, treatments’ adaptation, and culture consideration during recommendation making. The scale contains five responses, which span from strongly disagree to strongly agree. CSS was used to measure the cultural sensitivity of the nurses involved in this study. Nurses who scored the mean value or above were considered culturally sensitive, while those who scored less than the mean value were considered not culturally sensitive. ICS is a 5-point Likert scale, and the scale contains five items asking the nurses to rate their ability to communicate, comprehend points of view, resolve misunderstandings, and empathize with patients while interacting with patients from diverse cultural backgrounds. Items on this scale were rated using five responses spanning from strongly disagree to strongly agree. The total scale score is between 5 and 25. ICS was used to measure the intercultural communication ability of nurses who participated in this study. Nurses who scored the mean value or above were considered to have effective intercultural communication ability, while those who scored less than the mean value were considered to have ineffective intercultural communication ability.

#### Interpersonal communication scale (IPCS)

The interpersonal communication ability of nurses who participated in this study was measured using a previously published scale called IPCS. IPCS was previously developed and published online by Campbell & Atas Akdemir (2016) [22]. The scale consists of seven items, with responses ranging from strongly disagree to strongly disagree. Nurses who scored the mean value or above were considered to have effective interpersonal communication ability, while those who scored less than the mean value were considered to have ineffective interpersonal communication ability.

#### Cultural Motivation Scale (CMS)

The cultural motivation of nurses involved in this study was measured by the cultural motivation scale derived from the cultural motivation subscale of the cultural intelligence scale, which was first developed and published online by Ang & Van Dyne (2008b) [23]. CMS is a 5-point Likert scale that contains five items, with responses ranging from strongly disagree to strongly agree. The total score for CMS is between 5 and 25. Nurses who scored the mean value or above were considered to be culturally motivated, while those who scored less than the mean value were considered not to be culturally motivated.

### Statistical analysis

Data were exported into Statistical Product and Service Solution (SPSS) Version 26.0 for analysis. It was then analyzed using descriptive statistics, such as mean scores and standard deviations, and inferential statistics. All variables were entered into bivariate binary logistic regression analysis, and predictor variables with $$ p<$$0.25 were considered potential candidates for multivariable logistic regression analysis. In the multivariable logistic regression model, $$p\leq$$0.05 and adjusted odds ratio were used to declare statistical significance and strength of association. Binary logistic regression assumptions were checked before data analysis. Multi-collinearity was checked to see the linear correlation among the independent variables by using the variance inflation factor and tolerance test. All of the variables had a variance inflation factor of less than 10. Hosmer and Lemeshow’s test was found to be insignificant (*p*=0.750) and the Omnibus test was significant (p$$ <$$0.001), indicating that the model was fitted.

## Results

Out of the total sample, 236 nurses participated in this study, giving a response rate of 96.72%.

### Respondents’ demographic characteristics

The majority of the studied nurses (75.0%) were in the age group of 25–29 years. The mean age of the respondents was 27.53 years (SD $=\pm$3.13 years). More than half (55.9%) of them were female and the majority of them (42.8%) were orthodox religion followers. More than half of the studied nurses (58.5%) were Oromo in ethnicity. The vast majority of them (80.5%) were B.Sc. degree holders. More than two-thirds (68.2%) of the respondents had a monthly income of less than 6,000 ETB. Regarding professional experience, most of the studied nurses (80.9%) reported that they served for less than 5 years [Table 2].

**Table 2:** Demographic Characteristics of Nurses in Jimma Medical Center, Oromia Regional State, Southwest Ethiopia, May 20th to June 20th, 2020 (*n* = 236)

Variables and categories		Frequency	Percentage (%)
**Age (years)**	$$ <$$25 25–29 $$ \ge $$30	17 177 42	7.2 75.0 17.8
**Sex**	Female Male	132 104	55.9 44.1
**Ethnicity**	Amhara Oromo Dawro Other*	77 138 12 9	32.6 58.5 5.1 3.8
**Religion**	Protestant Orthodox Muslim Wakeffata	68 101 66 1	42.8 28.0 28.8 0.4
**Level of education**	Diploma B.Sc. and above MSc	40 190 6	16.9 80.6 2.5
**Monthly income**	$$ <$$6000 ETB $$ \ge $$ 6,000 ETB	161 75	68.2 31.8
**Professional experience**	$$ <$$ 5 years $$ \ge $$ 5 years	191 45	80.9 19.1
**Current role**	Supervisor nurse Head nurse Staff nurse	5 8 223	2.1 3.4 94.5

*Gurage, Kafa, Sidama, Wolayta, Tigrie

### Cultural sensitivity

**Fig. 1:**
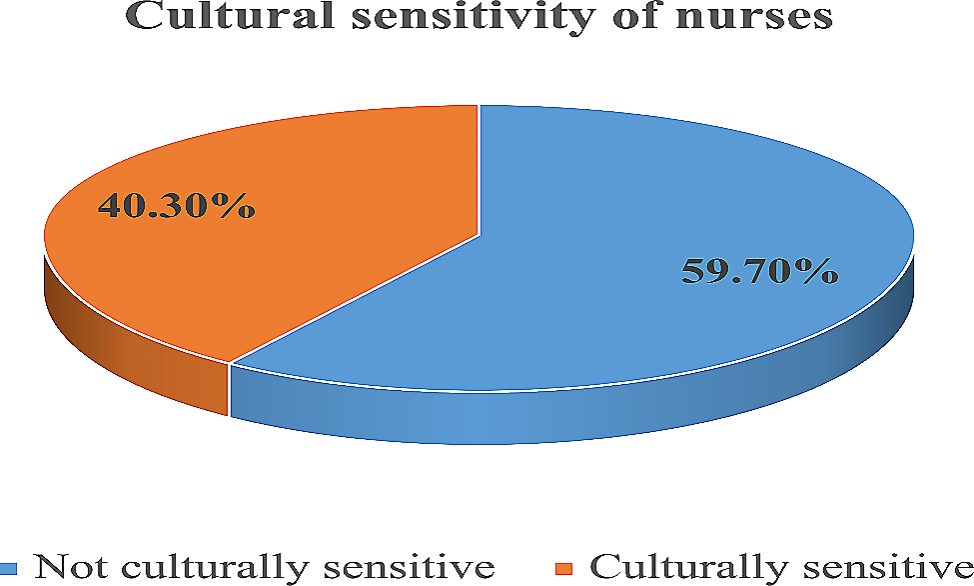
Cultural sensitivity among nurses in Jimma Medical Center, Oromia Regional State, Southwest Ethiopia, May 20th to June 20th, 2020 (*n* = 236)

Out of the participants involved in this study, 40.3% (95) of nurses were found to be culturally sensitive while delivering routine nursing services **[**Fig. 1**].**

### Factors affecting cultural sensitivity

Bivariate binary logistic regression analyses were used to select candidate variables for multivariable linear regression analysis. After bivariate analyses were carried out, seven variables were selected and entered into a multivariable binary logistic regression model using backward elimination to see their association with cultural sensitivity. From the variables entered into the final model, the multivariable binary logistic regression model revealed that the level of education, interpersonal communication, and intercultural communication were significantly associated with the cultural sensitivity of nurses. Accordingly, nurses with BSc degree and above were about 5 times more likely to be culturally sensitive when compared to diploma-holder nurses [AOR (95% CI)], [4.846 (1.188, 19.773)]. Additionally, nurses with effective interpersonal communication were also about 5 times more likely to be culturally sensitive while delivering care when compared to their counterparts [AOR (95% CI)], [4.749 (1.334, 16.909)]. Furthermore, nurses with effective intercultural communication were 52 times more likely to be culturally sensitive while delivering care when compared to those with ineffective intercultural communication [AOR (95% CI)], [51.874 (13.768, 195.45)] [Table 3].

**Table 3:** Bivariate and multivariable binary logistic regression results showing factors associated with cultural sensitivity among nurses in Jimma Medical Center, Oromia Regional State, Southwest Ethiopia, May 20th to June 20th, 2020 (*n* = 236)

Variables and category		Cultural sensitivity		COR (95%CI)	AOR (95%CI)
		Sensitive	Not sensitive		
**Sex**	**Female**	46 (30.3%)	106 (69.7%)	**1**	
	**Male**	49 (58.3%)	35 (41.7%)	3.226 (1.852,5.619)	1.313 (0.456, 3.783)
**Age**	$$ <$$ **30 years**	62 (32%)	132 (68%)	1	
	$\ge$ **30 years**	33 (78.6%)	9 (21.4%)	7.806 (3.520, 17.312)	3.186(1.153,8.803)
**Education**	**Diploma**	5 (12.5%)	35 (87.5%)	**1**	
	**BSc and above**	90 (45.9%)	106 54.1%)	5.943 (2.235, 15.808)	4.846 (1.188, 19.773)*
**Professional experience**	$$ <$$ **5 years**	58 (30.4%)	133 (69.6%)	**1**	
	$\ge$ **5 years**	37 (82.2%)	8 (17.8%)	10.606 (4.652, 24.179)	2.994(1.004,8.932)
**Interpersonal communication**	**Ineffective**	10 (7.9%)	116 (92.1%)	**1**	
	**Effective**	85 (77.3%)	25 (22.7%)	39.440 (17.991, 86.462)	4.749 (1.334, 16.909)*
**Cultural motivation**	**Not motivated**	22 (17.9%)	101 (82.1%)	**1**	
	**Motivated**	73 (64.6%)	40 (35.4%)	8.378 (4.593, 15.283)	0.879 (0.285, 2.710)
**Intercultural communication**	**Ineffective**	4 (3.2%)	121 (96.8%)	**1**	
	**Effective**	91 (18%)	20 (82%)	137.637 (45.48, 416.57)	51.874 (13.768, 195.45)*

Dependent variable: Cultural sensitivity; *=*p*-value = or < 0.05: 1 = Reference, COR = Crude odds ratio; AOR = Adjusted odds ratio; CI = Confidence interval

## Discussion

Nursing profession is holistic in its nature. Hence, it must consider patients’ cultural backgrounds in addition to other patient characteristics. Improving the cultural sensitivity of nurses is extremely important for the provision of culturally competent care. In the current study, cultural sensitivity among nurses and its associated factors were investigated. The finding shows that 40.3% (95% CI (34.3, 46.6)) of nurses who participated in this study were found to be culturally sensitive while providing care to patients of different cultural backgrounds, and this finding is similar to the cultural sensitivity of community health nurses reported in Southern Taiwan (39.53%) [25]. Conversely, this finding is lower than the cultural sensitivity of nurses reported in Turkey (69.6%) [19]. Other studies conducted in Turkey reported that the mean scores for cultural sensitivity of nurses are high [16, 26, 27], and their findings are much higher than the finding reported in our study. Variations in the reported levels of cultural sensitivity may be explained by the differences in data collection tools, geographical location, patient population, and healthcare system characteristics. Recognizing these factors is essential for interpreting and generalizing findings across different populations and settings. It is also crucial for a nurse to gather information about the patients’ cultural background. If a nurse does not gather cultural information, he or she will not be able to comprehend the patient’s behaviour or ascertain the cause of the patient’s refusal of medical therapy [28].

In our findings, it has also been shown that nurses with BSc degree and above are more likely to be culturally sensitive than diploma-holder nurses. This is supported by the studies conducted in south-central and western Turkey, in which nurses with higher educational levels demonstrated higher intercultural sensitivity levels [16, 26]. In the literature, higher scores of cultural sensitivity were obtained by nurses who had taken part in in-service education; this shows that cultural sensitivity is positively impacted by transcultural nursing courses and in-service education [26]. The possible reason for these similarities across literature could be due to the fact that higher levels of education promote critical thinking skills, which are invaluable in navigating complex cultural situations in healthcare. Therefore, global healthcare agendas that call for clinical and formal education should include the information and abilities needed to guarantee that nursing care is culturally sensitive. Furthermore, it is suggested that continuous education be mandated for all practicing nurses because education improves cultural sensitivity through teaching strategies [29, 30].

When providing care for patients from different cultural origins, nurses face numerous problems [31]. In our study, it has been indicated that nurses’ effective interpersonal and intercultural communication abilities determine their cultural sensitivity. Literature indicates that communication and culture are closely intertwined. Communication is the means by which culture is transmitted and preserved [32]. Nurses with effective intercultural communication have higher cultural sensitivity scores because they are better equipped to navigate potential barriers, such as language differences, non-verbal cues, and cultural expectations. Therefore, nurses should be given the opportunity to acquire cross-cultural communication skills as part of their graduate education and lifelong learning programs to help them comprehend and interact with people from many cultural backgrounds [30]. Effective communication with patients from different cultural backgrounds is essential for mutual understanding and for providing the best possible care [33]. From another point of view, ineffective intercultural communication can lead to inadequate comprehension of health issues, treatment plans, guidance, and instructions; suboptimal care quality and inappropriate interventions; and a decline in the relationship between nurses and patients [34–36]. By offering culturally sensitive care, nurses can understand both the similar and different perceptions and thoughts of patients. This helps them become more adept at providing individual-centered health care by helping them understand how patients’ values and beliefs impact the nurse-patient relationship. A nurse must respond sensitively and possess individual knowledge of cultural differences and similarities to provide culturally appropriate care [37].

Evidence generated in this study supports the implementation of necessary changes in nursing education and practice. Although the study provided valuable information on cultural sensitivity in a multicultural society, it is prone to several limitations. First, social desirability bias could have been introduced as the study relied on self-described information. Second, the generalization of the study’s findings may not apply to nurses working at private hospitals. Finally, the cross-sectional nature of the study constrains the ability to declare cause-and-effect relationships between the outcome variable and covariates.

### Implications for nursing practice and education

Cultural sensitivity in nursing practice has significant implications for healthcare outcomes, patient satisfaction, and the overall quality of care. Cultural sensitivity is a fundamental aspect of cultural competence. When nursing education fails to emphasize the importance of cultural sensitivity, students may graduate without developing the necessary skills and knowledge to effectively interact with and care for patients from diverse cultural backgrounds. This can result in a lack of understanding and appreciation for cultural differences, leading to suboptimal patient care. Additionally, cultural sensitivity promotes respect for diverse beliefs, values, and practices. Culturally sensitive nurses recognize and appreciate the differences among patients, avoiding stereotypes and biases. This fosters an inclusive environment where patients feel valued and respected. Furthermore, low cultural sensitivity among nurses can contribute to health disparities. Patients from marginalized or minority communities may face unique health challenges and have distinct healthcare needs. If nurses are not culturally sensitive, they may overlook or dismiss these needs, resulting in disparities in access to care, treatment outcomes, and health equity. Finally, patients who perceive a lack of cultural sensitivity from healthcare providers may have negative experiences, feeling that their values, beliefs, and cultural practices are not respected. This can lead to decreased patient satisfaction, reduced adherence to treatment plans, and reluctance to seek healthcare services in the future. Ultimately, it can undermine the patient-provider relationship and hinder the delivery of patient-centered care.

## Conclusion

Cultural sensitivity is found to be low in the study area. A low level of cultural sensitivity can have significant negative implications for both nurses and patients. It can indicate limited cultural competence among nurses and decreased patient satisfaction because patients may feel disrespected, marginalized, or misunderstood. Increasing level of education, effective interpersonal and intercultural communication abilities positively predict the cultural sensitivity of nurses providing nursing care to patients of different cultures. The findings of this study emphasize that it is helpful for nurses to improve their knowledge of transcultural nursing theories and cultural understanding. Nurses must get organized training and ongoing education in cultural sensitivity to achieve the best possible health outcomes in countries like Ethiopia where ethnic and cultural diversity is growing. Offering training in cultural sensitivity during undergraduate or postgraduate education programs will move the nursing profession to a better position in Ethiopia as well as in the rest of the world while also improving patient care. Furthermore, future researchers are encouraged to conduct longitudinal studies to determine the sequence of events between cultural sensitivity and different independent variables. As a result, healthcare quality could be improved and health disparities may be avoided.

### Electronic supplementary material

Below is the link to the electronic supplementary material.

Supplementary Material 1

## Data Availability

The datasets used and analyzed in this study are available from the corresponding author upon reasonable request.

## Abbreviations

AOR: Adjusted odds ratio
CI: Confidence interval
CMS: Cultural motivation scale
COR: Crude odds ratio
CSS: Cultural sensitivity scale
ICS: Intercultural communication scale
IPCS: Interpersonal communication scale
JMC: Jimma Medical Center
SD: Standard deviation
SPSS: Statistical product and service solution
