# Supplementary material for: Cultural sensitivity and associated factors among nurses in southwest Ethiopia: a cross-sectional study
Source: BMC Nurs. 2024 Mar 15;23:182. doi: 10.1186/s12912-024-01838-8 (PMC10941586; doi:10.1186/s12912-024-01838-8)
Supplement: Supplementary file 1 — Supplementary Material 1 [file 12912_2024_1838_MOESM1_ESM.pdf]

**Intercultural communication scale (ICS)**

| S/N | Items | Strongly disagree | Disagree | Neutral | Agree | Strongly agree |
|---|---|---|---|---|---|---|
| **301** | I understand the feelings of patients from other cultures | 1 | 2 | 3 | 4 | 5 |
| **302** | I communicate well with patients from other cultures | 1 | 2 | 3 | 4 | 5 |
| **303** | I can easily resolve misunderstandings with patients from other cultures | 1 | 2 | 3 | 4 | 5 |
| **304** | I understand the point of view of patients from other cultures | 1 | 2 | 3 | 4 | 5 |
| **305** | I can empathize with patients from other cultures | 1 | 2 | 3 | 4 | 5 |

**Cultural sensitivity scale (CSS)**

| S/N | Items | Strongly disagree | Disagree | Neutral | Agree | Strongly agree |
|---|---|---|---|---|---|---|
| **401** | I know a lot about my patients' culture | 1 | 2 | 3 | 4 | 5 |
| **402** | I adapt my treatment according to patients' culture | 1 | 2 | 3 | 4 | 5 |
| **403** | I consider patients' culture when making recommendations | 1 | 2 | 3 | 4 | 5 |

**Interpersonal communication scale (IPCS)**

| S/N | Items | Strongly disagree | Disagree | Neutral | Agree | Strongly agree |
|---|---|---|---|---|---|---|
| **501** | I encourage others to tell me how they feel | 1 | 2 | 3 | 4 | 5 |
| **502** | People tell me that I am easy to talk to | 1 | 2 | 3 | 4 | 5 |
| **503** | Strangers often approach and start talking to me | 1 | 2 | 3 | 4 | 5 |
| **504** | People tell me I am a good listener | 1 | 2 | 3 | 4 | 5 |
| **505** | I am honest with others about my thoughts and feelings | 1 | 2 | 3 | 4 | 5 |
| **506** | I believe that communication will be productive | 1 | 2 | 3 | 4 | 5 |
| **507** | I use examples to help me explain what I am talking about | 1 | 2 | 3 | 4 | 5 |

**Cultural motivation scale**

| S/N | Items | Strongly disagree | Disagree | Neutral | Agree | Strongly agree |
|---|---|---|---|---|---|---|
| **601** | I enjoy interacting with people from different cultures | 1 | 2 | 3 | 4 | 5 |
| **602** | I am confident that I can socialize with locals in a culture that is unfamiliar to me | 1 | 2 | 3 | 4 | 5 |
| **603** | I am sure I can deal with the stresses of adjusting to a culture that is new to me | 1 | 2 | 3 | 4 | 5 |
| **604** | I enjoy living in cultures that are unfamiliar to me | 1 | 2 | 3 | 4 | 5 |

| | | | | | | |
|---|---|---|---|---|---|---|
| **605** | I am confident that I can get accustomed to the shopping conditions in a different culture | 1 | 2 | 3 | 4 | 5 |
